# *KRAS* and *BRAF* Mutations in 203 Esophageal Squamous Cell Carcinomas: Pyrosequencing Technology and Literature Review

**DOI:** 10.1245/s10434-012-2819-z

**Published:** 2012-12-30

**Authors:** Hironobu Shigaki, Yoshifumi Baba, Masayuki Watanabe, Keisuke Miyake, Asuka Murata, Shiro Iwagami, Takatsugu Ishimoto, Masaaki Iwatsuki, Naoya Yoshida, Hideo Baba

**Affiliations:** Department of Gastroenterological Surgery, Graduate School of Medical Science, Kumamoto University, Kumamoto, Japan

## Abstract

**Background:**

Epidermal growth factor receptor (EGFR) signaling is one of the most promising targets for molecular-targeted therapies in esophageal squamous cell carcinoma (ESCC). Thus, the molecular diagnosis of *KRAS* and *BRAF* mutations is clinically important in therapeutic decision making. However, the frequency of *KRAS* and *BRAF* mutations in ESCCs remains inconclusive because of the limited sample sizes of previous studies (all *N* ≤ 80). Pyrosequencing is a nonelectrophoretic nucleotide extension sequencing technology that can be used for mutation testing.

**Methods:**

The frequency of *KRAS* and *BRAF* mutations was examined using a nonbiased database of 203 resected ESCCs and a high-throughput pyrosequencing assay.

**Results:**

The validity of the *KRAS* pyrosequencing method was initially demonstrated by detection of all 4 types of *KRAS* mutations [c.35G>T (codon 12 GGT>GTT), c.35G>A (codon 12 GGT>GAT), c.34G>T (codon 12 GGT>TGT), c.38G>A mutation (codon 13 GGC>GAC)], which had been previously diagnosed using Scorpion-ARMS technology, in 9 colon cancer tissues (9 of 9; 100 %). Similar results were demonstrated for *BRAF* mutational status in 3 colon cancer cell lines (HCT116, Colo201, and HT29), which were validated by Sanger dideoxy sequencing. Subsequently, the *KRAS* mutation was found to be extremely rare (1 of 203; 0.5 %), and the *BRAF* mutation was absent (0 of 203; 0 %), in the dataset of 203 ESCCs.

**Conclusions:**

These results suggest that *KRAS* and *BRAF* mutations play a limited role in the development of ESCC and that mutation analysis is not useful as a screening test for sensitivity to anti-EGFR therapy in ESCC.

Esophageal squamous cell carcinoma (ESCC) is the major histological type of esophageal cancer in East Asian countries and is one of the most aggressive malignant tumors.^1^ Despite remarkable advances in multimodal therapies, patient prognosis remains poor, even for those whose carcinomas have been completely resected.^2^–^5^ The limited improvement in treatment outcomes by conventional therapies urged us to seek innovative strategies for treating ESCC, especially those that are molecularly targeted. One of the most promising targets is the inhibition of the epidermal growth factor receptor (EGFR) by monoclonal antibodies (e.g., cetuximab, panitumumab) or small molecule tyrosine kinase inhibitors (e.g., erlotinib, gefitinib).^6^–^10^ The EGFR signal transduction network plays a crucial role in multiple tumorigenic processes, contributing to cell-cycle progression, angiogenesis, metastasis, and protection of the cancer cell from apoptosis.^11^ Mutations in the Kirsten Ras 1 (*KRAS*) and V-Raf Murine Sarcoma Viral Oncogene Homolog B1 (*BRAF*) genes may be predictive of response to drugs directly linked to the EGFR pathway.^12^–^14^ Thus, molecular diagnosis of these mutations is increasingly important in making therapeutic decisions. Several previous studies have examined the frequency of *KRAS* and *BRAF* mutations in ESCCs; however, they were all limited by small sample sizes (all *N* ≤ 80) (Table 1), yielding inconclusive results.^8^,^15^–^20^

**Table 1:** Studies on KRAS and BRAF mutations in ESCC

Study	Sample size	Methods	Mutation detected *N* (%)	Codons examined
Studies on KRAS mutations in ESCC				
Ma et al.^15^	35	Pyrosequencing	2 (5.7 %)	Codons 12–13
Liu et al.^16^	50	Pyrosequencing	6 (12 %)	Codons 12–13
Lorenzen et al.^8^	37	Direct sequencing	0 (0 %)	Codons 12–13
Hollstein et al.^17^	16	Direct sequencing	0 (0 %)	Codons 12–13
Victor et al.^18^	27	PCR and oligomer hybridization assay	0 (0 %)	Codons 12–13
Hollstein et al.^19^	25	PCR and oligomer hybridization assay	0 (0 %)	Codons 12–13
Present study	203	Pyrosequencing	1 (0.5 %)	Codons 12–13

Studies on BRAF mutations in ESCC				
Ma et al.^15^	35	Pyrosequencing	0 (0 %)	Codon 600
Maeng et al.^20^	80	OncoMap	1 (1.2 %)	Codon 600
Present study	203	Pyrosequencing	0 (0 %)	Codon 600

Pyrosequencing is a nonelectrophoretic nucleotide extension sequencing technology for various applications, including mutation testing of tumors.^21^–^24^ This technology has several advantages. First, it has higher sensitivity than classical Sanger dideoxy sequencing.^22^ Sanger dideoxy sequencing needs more than 20 % of tumor load in a specimen to render a reliable result, while pyrosequencing can render a reliable result with a tumor load of 5 %. Second, pyrosequencing is faster than Sanger dideoxy sequencing. Third, pyrosequencing is more cost effective. Collectively, pyrosequencing is a useful method in molecular diagnostics and large-scale epidemiological studies.^25^,^26^

Therefore, in the present study, *KRAS* and *BRAF* mutations were screened using a nonbiased database of 203 ESCCs and pyrosequencing technology.

## Patients and Methods

### Study Subjects

A total of 217 consecutive patients with ESCC who were undergoing curative resection at Kumamoto University Hospital between April 2005 and December 2010 were enrolled in this study. There were 13 patients excluded because of the unavailability of adequate tissue samples. We initially quantified *KRAS* and *BRAF* mutation in 204 cancer specimens and obtained valid results in 203 cases (99.5 %). Thus, 203 ESCCs were finally included in this study. Tumor staging was done by the American Joint Committee on Cancer Staging Manual (7th edition).^27^ Written informed consent was obtained from each subject, and the institutional review board of Kumamoto University approved the study procedures.

A total of 9 patients with colon cancers harboring 4 different *KRAS* mutations [c.35G>T (codon 12 GGT>GTT; p.Gly12Val), c.35G>A (codon 12 GGT>GAT; p.Gly12Asp), c.34G>T (codon 12 GGT>TGT; p.Gly12Cys), and c.38G>A mutation (codon 13 GGC>GAC; p.Gly13Asp)], which had been already diagnosed by Scorpion-ARMS technology, were also included in this study to validate the pyrosequencing method for the detection of *KRAS* mutations.

### Genomic DNA Extraction

One pathologist marked the tumor areas on slides stained with hematoxylin-eosin. Genomic DNA was extracted from tumor lesions enriched with neoplastic cells, without adjacent normal tissue, using an FFPE kit (Qiagen, Valencia, CA). DNA was also extracted from 3 cell lines: Colo201 and HT29 with the *BRAF* mutation c.1799T>A (p.V600E), and HCT116 with wild-type *BRAF* using a QIAmp DNA mini kit (Qiagen, Valencia, CA).^28^,^29^ DNA was stored at −20 °C before use.

### Whole Genome Amplification

Whole genome amplification (WGA) is a useful technique for preserving original study material for many different assays and for future studies. In WGA, genomic DNA is amplified by polymerase chain reaction (PCR) using primers consisting of a random sequence of 15 nucleotides. Each PCR mix contained 40 pmol of the random primers, 1.0 nmol each of dNTP, 2.0 mmol/L MgCl2, 1× PCR buffer (Applied Biosystems, Foster City, CA), 0.25 U of AmpliTaq Gold 360 (Applied Biosystems), and 5 μl of template DNA solution in a total volume of 50 μl. PCR conditions consisted of initial denaturation at 95 °C for 10 min; 50 cycles of 95 °C for 60 s, annealing (37 °C for 2 min), ramping from 37 to 55 °C (0.1 °C/s), 55 °C for 2 min, and 68 °C for 30 s; and a final extension at 72 °C for 7 min.

### Pyrosequencing for KRAS and BRAF Mutations

Pyrosequencing technology has been shown to reliably detect *KRAS* mutations with 100 % analytic sensitivity and specificity, even when the proportion of mutant alleles is as low as 10 %. PCR amplification primers for pyrosequencing targeted for *KRAS* (codons 12, 13), *BRAF* (codon 600) were: KRAS-F, forward, 5′-NNNGGCCTGCTGAAAATGACTGAA-3′; and KRAS-R, reverse biotinylated primer, 5′-TTAGCTGTATCGTCAAGGCACTCT-3′; and BRAF-F, forward biotinylated primer, 5′-CAGTAAAAATAGGTGATTTTG-3′; and BRAF-R reverse, 5′-TCCAGACAACTGTTCAAACTGA-3′. Each PCR mix contained the forward and reverse primers (20 pmol each), 1.0 nmol of each dNTP with dUTP, 2 mmol/L MgCl2, 1× PCR buffer, 1.25 U of AmpliTaq Gold 360, 0.5 U of AmpErase UNG and 5 μl of template WGA product in a total volume of 50 μl. PCR condition consisted of initial denaturation at 50 °C (10 min) for AmpErase UNG; initial denaturation at 94 °C (10 min) for AmpliTaq Gold 360; 50 cycles of 95 °C (30 s), annealing (30 s; 55 °C for BRAF, 57 °C for KRAS), and 72 °C (30 s); and a final extension at 72 °C (7 min). The PCR products were electrophoresed through an agarose gel to confirm successful amplification of the 82-bp (*KRAS*) and the 80-bp PCR (*BRAF*) product.

**Fig. 1:**
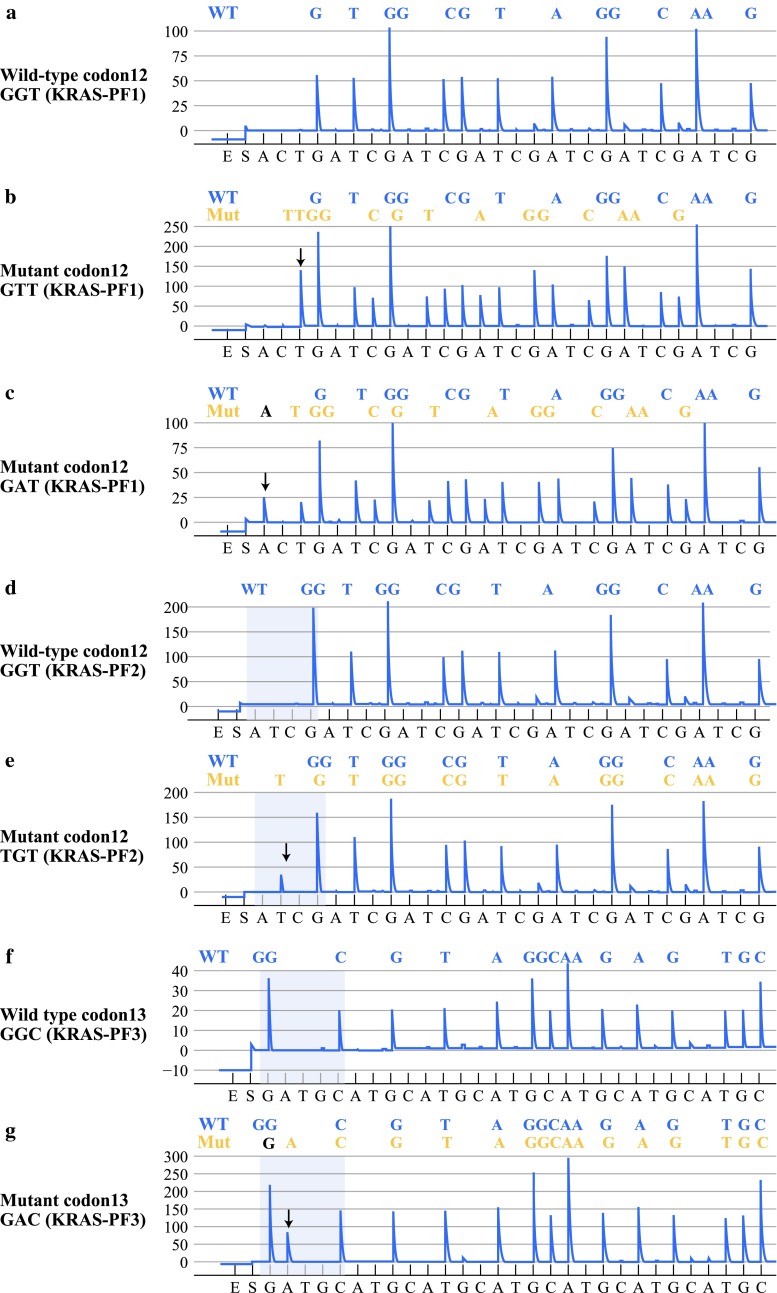
Pyrograms of wild-type and mutant KRAS in colorectal carcinoma. **a** Wild-type codon 12 detected by the KRAS-PF1 primer. **b** c.35GT (codon 12 GTT) mutation detected by the KRAS-PF1 primer. **c** c.35GA (codon 12 GAT) mutation detected by the KRAS-PF1 primer. **d** Wild-type codon 12 detected by the KRAS-PF2 primer. **e** c.34GT (codon 12 TGT) mutation detected by the KRAS-PF2 primer. **f** Wild-type codon 13 detected by the KRAS-PF3 primer. **g** c.38GA (codon 13 GAC) mutation detected by the KRAS-PF3 primer. *Arrows* indicate the presence of mutant alleles

**Fig. 2:**
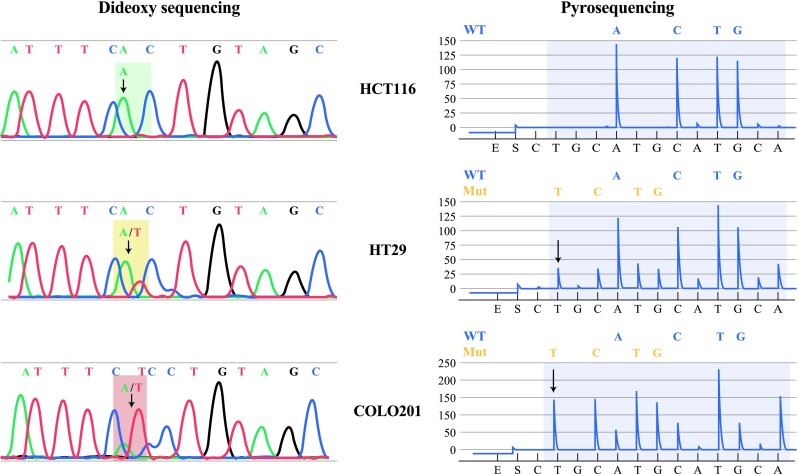
Detection of BRAF V600E in colon cancer cell lines by dideoxy sequencing and pyrosequencing; detection of homozygous wild type (HCT116), heterozygous mutant (HT29, COLO201). BRAF V600E variants identified by dideoxy sequencing (*left panel*) and pyrosequencing (*right panel*). The pyrosequencing nucleotide dispensation order is shown below each pyrogram. The numerical position for each nucleotide is indicated at the *top*. *Arrows* indicate the presence of mutant alleles

*KRAS* and *BRAF* pyrosequencing was performed using the PyroMark Q24 System (Qiagen, Valencia, CA) according to the manufacturer’s instructions (Fig. 1 for *KRAS*; Fig. 2 for *BRAF*). All forward sequencing results were confirmed by reverse sequencing. In the *KRAS* pyrosequencing assay, the presence of a mutation was routinely confirmed by 3 different sequencing primers and by the creation of the frameshifted open reading frame of the mutant sequence relative to a wild-type sequence in a program. The primer KRAS-PF1 (5′-TGTGGTAGTTGGAGCTG-3′; pyrosequencing nucleotide dispensation order, ACTGATCG ATCGATCGATCGATCGATCG) could detect the c.35G>T (codon 12 GTT) and c.35G>A (codon 12 GAT) mutations. The primer KRAS-PF2 (5′-TGTGGTAGTTGGAGCT-3′; pyrosequencing nucleotide dispensation order, ATCGATCGATCGATCGATCGATCATCG) could detect the c.34G>T (codon 12 TGT) mutation. The primer KRAS-PF3 (5′-TGGTAGTTGGAGCTGGT-3′; pyrosequencing nucleotide dispensation order, GATGCATGCATGCATGCATGCATGCATGC) could detect the c.34G>A (codon 13 GAC) mutation. In the BRAF pyrosequencing assay, the primer was 5′-CACTCCATCGAGATTTC-3′, and the pyrosequencing nucleotide dispensation order was CTGCATGCATGCTGCA.

## Results

### Clinical Data

A summary of the clinical characteristics of the patients is given in Table 2. In this cohort, the 5-year overall survival rates of patients treated by esophagectomy were 83.9 % for stage I, 59.7 % for stage II, and 36.7 % for stage III. These rates are similar to those from the “Comprehensive Registry of Esophageal Cancer in Japan” (79.5 % for stage I, 58.9 % for stage II, and 39.8 % for stage III), which supports the absence of bias in our database.^30^

**Table 2:** Patient characteristics

Clinical characteristics	Total (*N*)
All cases	203
Mean age ± SD	66.8 ± 9.24
Sex	
Male	178 (87.7 %)
Female	25 (12.3 %)
Preoperative treatment	
Present	68 (33.5 %)
Absent	135 (66.5 %)
Cancer location	
Upper thoracic	31 (15.3 %)
Middle thoracic	105 (51.7 %)
Lower thoracic	58 (28.6 %)
Esophagogastric junction	9 (4.4 %)
Stage	
I (IA, IB)	76 (37.4 %)
II (IIA, IIB)	65 (32.0 %)
III (IIIA, IIIB, IIIC)	62 (30.5 %)
Lymph node metastasis	
Positive	94 (46.3 %)
Negative	109 (53.7 %)
Histological grade	
G1	83 (40.9 %)
G2	82 (40.4 %)
G3–4	28 (13.8 %)

### Validation of the Pyrosequencing Assay for KRAS Mutation Detection

We first examined the validity of the pyrosequencing method using eleven colon cancer tissues harboring 4 different *KRAS* mutations [c.35G>T (codon 12 GGT>GTT; p.Gly12Val), c.35G>A (codon 12 GGT>GAT; p.Gly12Asp), c.34G>T (codon 12 GGT>TGT; p.Gly12Cys), and c.38G>A mutation (codon 13 GGC>GAC; p.Gly13Asp)]. These mutations had already been diagnosed by the sensitive Scorpion-ARMS technology [i.e., Amplification Refractory Mutation System incorporating a unique bifunctional florescent primer/probe molecule (Scorpion)]. As shown in Table 3, all 4 types of mutations could be detected using the pyrosequencing technology (11 of 11, 100 %) (Fig. 1), which demonstrated that the method was reliable for the detection of *KRAS* mutations in tumors.

**Table 3:** Comparative analysis of Scorpion-ARMS and pyrosequencing for detection of KRAS mutation in 9 colorectal carcinoma tissues

*KRAS* mutation type	Results of Scorpion-ARMS (*n*)	Results of pyrosequencing (*n*)	Concordance rate
Codon12 GAT	3	3	100 % (3/3)
Codon12 GTG	2	2	100 % (2/2)
Codon12 TGT	2	2	100 % (2/2)
Codon13 GAC	2	2	100 % (2/2)
Total	9	9	100 % (9/9)

### KRAS Mutation in ESCC

Pyrosequence analysis of *KRAS* codon 12 and 13 mutations was successful for 203 of 204 (99.5 %) ESCC paraffin-embedded tissues. Among the 203 ESCCs, only 1 case harbored a *KRAS* mutation [c.34G>T (codon 12 GGT>TGT; p.Gly12Cys)]. This mutation was also diagnosed by the Scorpion-ARMS technology (data not shown). This result showed that the frequency of *KRAS* mutations in ESCC was extremely low.

### Validation of Pyrosequencing Assay for BRAF Mutation Detection

To validate our *BRAF* pyrosequencing assay, both pyrosequencing and Sanger dideoxy sequencing were performed on the same set of DNA samples from 3 colon cancer cell lines (HCT116, Colo201, and HT29). HCT116 possesses wild-type *BRAF*, and Colo201 and HT29 harbors a *BRAF* mutation [c.1799T>A (p.V600E)]. In Sanger dideoxy sequencing, HCT116 showed was homozygous wild-type for the *BRAF* gene, and HT29 and COLO201 were shown to be heterozygous *BRAF* V600E mutants (Fig. 2). Among all 3 cell lines, pyrosequencing gave the same results as Sanger sequencing for *BRAF* mutational status (Fig. 2).

Furthermore, the *BRAF* mutation could be detected in paraffin-embedded tissues of colon cancer that had been previously diagnosed by Sanger dideoxy sequencing. Collectively, these preliminary experiments supported the validity of the pyrosequencing method for the detection of *BRAF* mutation in paraffin-embedded specimens.

### BRAF Mutation in ESCC

The mutational status in *BRAF* exon 15 (V600E) was examined in 204 ESCCs, and valid results were obtained in 203 tissues (99.5 %). All 203 ESCCs were wild-type at codon 600 in *BRAF* exon 15.

## Discussion

*KRAS* and *BRAF* mutational status could represent a predictive marker for anti-EGFR therapies; therefore, better understanding of the incidence of these mutations is important. However, the frequency of *KRAS* and *BRAF* mutations in ESCCs remains inconclusive because of the limited sample sizes of previous studies.^8^,^15^–^20^ The validity of the pyrosequencing method for detecting *KRAS* and *BRAF* mutations was initially demonstrated using colon cancer cell lines and colon cancer tissues harboring *KRAS* and *BRAF* mutations. Thereafter, *KRAS* mutations were shown to be extremely rare in a database of more than 200 ESCCs. In addition, the *BRAF* mutation was absent in ESCC tumors.

Pyrosequencing is a nonelectrophoretic nucleotide extension sequencing technology that can be used for mutation detection in tumors.^21^–^24^ Pyrosequencing offers a higher sensitivity than classical Sanger dideoxy sequencing for the detection of *KRAS* mutations.^22^ In addition, because of its simplicity and cost effectiveness, pyrosequencing represents a potentially useful method in molecular diagnostics and epidemiological studies, particularly in the setting of large-scale projects and clinical assays.^25^,^26^

Mutations in the *KRAS* gene occur early in the development of several types of cancers.^31^ Commonly restricted to codon 12 and 13 in exon 2, these mutations cause impaired GTPase activity and result in a continual stimulus for cellular proliferation. They have been found in more than 40 % of colorectal cancers, 90 % of pancreatic carcinomas, and 33 % of non-small-cell lung carcinomas.^32^ Importantly, *KRAS* mutation status has recently become an important biomarker when identifying resistance to anti-EGFR therapy. Several previous studies have examined the frequency of *KRAS* mutations in ESCCs; only 2 studies (*N* = 35 and 50) showed the presence of *KRAS* mutations in ESCCs; however, others have demonstrated the absence of *KRAS* mutation in ESCCs.^8^,^15^–^19^ Unfortunately, all previous studies were limited by small sample sizes (*N* ≤ 80). It should be noted that small studies are more prone to “publication bias” than large studies. The phenomenon of publication bias occurs because studies with null findings (e.g., absence of *KRAS* mutation in tumors) have a higher likelihood of being unwritten and unpublished compared with those with significant results (e.g., presence of *KRAS* mutation in tumors). Compared with small studies with null data, large studies with null data are more likely to be published. As a result, large studies are less prone to publication bias than small studies. Publishing null data from well-powered studies are important because publishing significant results from small underpowered studies also leads to publication bias. In this respect, our finding that *KRAS* mutations are extremely rare in a nonbiased database of more than 200 ESCCs may have considerable implications.

The *BRAF* gene encodes a serine/threonine kinase of the RAS RAF MEK MAPK signaling pathway and is mutated in a variety of cancer types.^33^ The V600E point mutation in exon 15 of the *BRAF* gene has been shown to be associated with insensitivity to antigrowth signals, cell-cycle dysregulation, tumor invasion and metastasis, escape from apoptosis, unlimited replicative potential and angiogenesis, and can be used as a predictive biomarker for *BRAF*-targeted therapy. In addition, the prognostic role of the *BRAF* mutation has been emphasized in several types of cancers.^34^–^37^ However, the incidence of the *BRAF* mutation in ESCC remains less clear. One study showed the absence of a *BRAF* mutation in 35 ESCCs, and the other showed that only 1 tumor harbored a *BRAF* mutation among 80 ESCC tumors. In this study, the *BRAF* mutation was absent in 203 ESCCs.

In summary, *KRAS* mutations were extremely rare, and the *BRAF* mutation was absent in a nonbiased database of 203 ESCCs. This suggests that *KRAS* and *BRAF* mutations play a limited role in the development of ESCC and that mutation analysis is not useful as a predictive marker for sensitivity to anti-EGFR therapy in ESCC.

## References

[1] Enzinger PC, Mayer RJ (2003). Esophageal cancer. N Engl J Med..

[2] Thallinger CM, Raderer M, Hejna M (2011). Esophageal cancer: a critical evaluation of systemic second-line therapy. J Clin Oncol..

[3] Stahl M (2010). Is there any role for surgery in the multidisciplinary treatment of esophageal cancer?. Ann Oncol..

[4] Chen WH, Chao YK, Chang HK, Tseng CK, Wu YC, Liu YH (2012). Long-term outcomes following neoadjuvant chemoradiotherapy in patients with clinical T2N0 esophageal squamous cell carcinoma. Dis Esophagus..

[5] Pantling AZ, Gossage JA, Mamidanna R, Newman G, Robinson A, Manifold DK (2011). Outcomes from chemoradiotherapy for patients with esophageal cancer. Dis Esophagus..

[6] Martini M, Vecchione L, Siena S, Tejpar S, Bardelli A (2011). Targeted therapies: how personal should we go?. Nat Rev Clin Oncol..

[7] McNamara MJ, Adelstein DJ (2012). Current developments in the management of locally advanced esophageal cancer. Curr Oncol Rep..

[8] Lorenzen S, Schuster T, Porschen R, Al-Batran SE, Hofheinz R, Thuss-Patience P (2009). Cetuximab plus cisplatin-5-fluorouracil versus cisplatin-5-fluorouracil alone in first-line metastatic squamous cell carcinoma of the esophagus: a randomized phase II study of the Arbeitsgemeinschaft Internistische Onkologie. Ann Oncol..

[9] Ilson DH, Kelsen D, Shah M, Schwartz G, Levine DA, Boyd J (2011). A phase 2 trial of erlotinib in patients with previously treated squamous cell and adenocarcinoma of the esophagus. Cancer..

[10] Adelstein DJ, Rodriguez CP, Rybicki LA, Ives DI, Rice TW (2010). A phase II trial of gefitinib for recurrent or metastatic cancer of the esophagus or gastroesophageal junction. Invest New Drugs..

[11] Han W, Lo HW (2012). Landscape of EGFR signaling network in human cancers: biology and therapeutic response in relation to receptor subcellular locations. Cancer Lett..

[12] Walther A, Johnstone E, Swanton C, Midgley R, Tomlinson I, Kerr D (2009). Genetic prognostic and predictive markers in colorectal cancer. Nat Rev Cancer..

[13] Garnett MJ, Marais R (2004). Guilty as charged: B-RAF is a human oncogene. Cancer Cell..

[14] De Roock W, De Vriendt V, Normanno N, Ciardiello F, Tejpar S (2011). KRAS, BRAF, PIK3CA, and PTEN mutations: implications for targeted therapies in metastatic colorectal cancer. Lancet Oncol..

[15] Ma H, Xue Y, Li C, Zhang J, Ren Z (2011). A preliminary study on K-ras, EGFR, and B-raf mutations of esophageal squamous cell carcinoma. Chinese-German. J Clin Oncol..

[16] Liu QW, Fu JH, Luo KJ, Yang HX, Wang JY, Hu Y, et al. Identification of EGFR and KRAS mutations in Chinese patients with esophageal squamous cell carcinoma. *Dis Esophagus.* 2011. doi:10.1111/j.1442-2050.2010.01155.x. [Epub ahead of print]. 10.1111/j.1442-2050.2010.01155.x 21615826

[17] Hollstein MC, Peri L, Mandard AM, Welsh JA, Montesano R, Metcalf RA (1991). Genetic analysis of human esophageal tumors from two high incidence geographic areas: frequent p53 base substitutions and absence of ras mutations. Cancer Res..

[18] Victor T, Du Toit R, Jordaan AM, Bester AJ, van Helden PD (1990). No evidence for point mutations in codons 12, 13, and 61 of the ras gene in a high-incidence area for esophageal and gastric cancers. Cancer Res..

[19] Hollstein MC, Smits AM, Galiana C, Yamasaki H, Bos JL, Mandard A (1988). Amplification of epidermal growth factor receptor gene but no evidence of ras mutations in primary human esophageal cancers. Cancer Res..

[20] Maeng CH, Lee J, van Hummelen P, Park SH, Palescandolo E, Jang J (2012). High-throughput genotyping in metastatic esophageal squamous cell carcinoma identifies phosphoinositide-3-kinase and BRAF mutations. PLoS One..

[21] Fakhrai-Rad H, Pourmand N, Ronaghi M (2002). Pyrosequencing: an accurate detection platform for single nucleotide polymorphisms. Hum Mutat..

[22] Ogino S, Kawasaki T, Brahmandam M, Yan L, Cantor M, Namgyal C (2005). Sensitive sequencing method for KRAS mutation detection by Pyrosequencing. J Mol Diagn..

[23] Shen S, Qin D (2012). Pyrosequencing data analysis software: a useful tool for EGFR, KRAS, and BRAF mutation analysis. Diagn Pathol..

[24] Chen DC, Saarela J, Nuotio I, Jokiaho A, Peltonen L, Palotie A (2003). Comparison of GenFlex Tag array and Pyrosequencing in SNP genotyping. J Mol Diagn..

[25] Imamura Y, Morikawa T, Liao X, Lochhead P, Kuchiba A, Yamauchi M (2012). Specific mutations in KRAS codons 12 and 13, and patient prognosis in 1075 BRAF wild-type colorectal cancers. Clin Cancer Res..

[26] Ogino S, Meyerhardt JA, Irahara N, Niedzwiecki D, Hollis D, Saltz LB (2009). KRAS mutation in stage III colon cancer and clinical outcome following intergroup trial CALGB 89803. Clin Cancer Res..

[27] Rice TW, Blackstone EH, Rusch VW (2010). 7th edition of the AJCC Cancer Staging Manual: esophagus and esophagogastric junction. Ann Surg Oncol..

[28] Brandi G, Tavolari S, De Rosa F, Di Girolamo S, Agostini V, Barbera MA, et al. Antitumoral efficacy of the protease inhibitor gabexate mesilate in colon cancer cells harbouring KRAS, BRAF and PIK3CA mutations. *PLoS One.* 2012;7:e41347. 10.1371/journal.pone.0041347 PMC3404056 22911782

[29] Wang H, Daouti S, Li WH, Wen Y, Rizzo C, Higgins B (2011). Identification of the MEK1(F129L) activating mutation as a potential mechanism of acquired resistance to MEK inhibition in human cancers carrying the B-RafV600E mutation. Cancer Res..

[30] Ozawa S, Tachimori Y, Baba H, Fujishiro M, Matsubara H, Numasaki H (2012). Comprehensive registry of esophageal cancer in Japan, 2004. Esophagus..

[31] Loriot Y, Mordant P, Deutsch E, Olaussen KA, Soria JC (2009). Are RAS mutations predictive markers of resistance to standard chemotherapy?. Nat Rev Clin Oncol..

[32] Adjei AA (2001). Blocking oncogenic Ras signaling for cancer therapy. J Natl Cancer Inst..

[33] Davies H, Bignell GR, Cox C, Stephens P, Edkins S, Clegg S (2002). Mutations of the BRAF gene in human cancer. Nature..

[34] Ogino S, Shima K, Meyerhardt JA, McCleary NJ, Ng K, Hollis D (2012). Predictive and prognostic roles of BRAF mutation in stage III colon cancer: results from intergroup trial CALGB 89803. Clin Cancer Res..

[35] Ogino S, Nosho K, Kirkner GJ, Kawasaki T, Meyerhardt JA, Loda M (2009). CpG island methylator phenotype, microsatellite instability, BRAF mutation and clinical outcome in colon cancer. Gut..

[36] Grisham RN, Iyer G, Garg K, Delair D, Hyman DM, Zhou Q, et al. BRAF Mutation is associated with early stage disease and improved outcome in patients with low-grade serous ovarian cancer. *Cancer.* 2012. doi:10.1002/cncr.27782. [Epub ahead of print]. 10.1002/cncr.27782 PMC3961140 22930283

[37] Moreau S, Saiag P, Aegerter P, Bosset D, Longvert C, Hélias-Rodzewicz Z (2012). Prognostic value of BRAF (V600) mutations in melanoma patients after resection of metastatic lymph nodes. Ann Surg Oncol..

